# Pneumococcal Δ*pep27* Immunization Attenuates TLRs and NLRP3 Expression and Relieves Murine Ovalbumin-Induced Allergic Rhinitis

**DOI:** 10.4014/jmb.2203.03006

**Published:** 2022-04-18

**Authors:** Jae Ik Yu, Ji-Hoon Kim, Ki-El Nam, Wonsik Lee, Dong-Kwon Rhee

**Affiliations:** 1 School of Pharmacy, Sungkyunkwan University, Suwon 16419, Republic of Korea; 2 DNBio Pharm. Inc., Research Center, Sungkyunkwan University, Suwon 16419, Republic of Korea

**Keywords:** Δ*pep27*, allergic rhinitis, toll-like receptor, NLRP3 inflammasome

## Abstract

Allergic rhinitis (AR), one of the most common inflammatory diseases, is caused by immunoglobulin E (IgE)–mediated reactions against inhaled allergens. AR involves mucosal inflammation driven by type 2 helper T (Th2) cells. Previously, it was shown that the *Streptococcus pneumoniae* *pep27* mutant (Δ*pep27*) could prevent and treat allergic asthma by reducing Th2 responses. However, the underlying mechanism of Δ*pep27* immunization in AR remains undetermined. Here, we investigated the role of Δ*pep27* immunization in the development and progression of AR and elucidated potential mechanisms. In an ovalbumin (OVA)-induced AR mice model, Δ*pep27* alleviated allergic symptoms (frequency of sneezing and rubbing) and reduced TLR2 and TLR4 expression, Th2 cytokines, and eosinophil infiltration in the nasal mucosa. Mechanistically, Δ*pep27* reduced the activation of the NLRP3 inflammasome in the nasal mucosa by down-regulating the Toll-like receptor signaling pathway. In conclusion, Δ*pep27* seems to alleviate TLR signaling and NLRP3 inflammasome activation to subsequently prevent AR.

## Introduction

Allergic rhinitis (AR) is a significant health problem worldwide, and the incidence rate has increased over the past decades [1]. AR symptoms interfere with sleep, leading to a decrease in quality of life [2] and collapse in productivity and social functioning [3]. Patients with severe AR also have signs of significant anxiety, depression, and fatigue. AR has increased compared to the other allergic diseases including asthma and atopy [4]. The current therapeutic regimen does not cure AR but relieves it temporarily. Therefore, there is a need for the development of more effective treatment options. Nowadays, allergen-specific immunotherapy (AIT), which targets key molecules driving the Th2 response, is already used in the clinic, and a wave of novel drug candidates is under development [5]. AIT exhibits efficacy but is limited by high costs and time in AR clinical trials.

AR is mediated by Th2 cells, which release IL-4, IL-5, and IL-13. These cytokines lead to a series of events promoting B cell iso-type conversion with subsequent local and systemic allergen-specific IgE antibody production by plasma B cells and eosinophilic infiltration into the nasal epithelium [6]. Crosslinking of IgE, which is bound to mast cells by allergens, in turn, causes the release of mediators such as histamine and leukotriene, which are responsible for artery expansion, increased vascular permeability, itching, rhinorrhea, and mucous secretion [1].

Inactivated wild type pneumococci was reported to alleviate ovalbumin (OVA)-induced asthma [7]. However, due to the lethal nature of the wild type pneumococci, there could be some potential side effects of even the inactivated form. Moreover, specific TLR agonists might differentially activate innate immunity and induce AR and asthma in the nose and lungs, respectively. House dust mite (HDM)-derived β-glucans could activate TLR2 but not TLR4 and trigger AR in the nasal mucosa. However, the TLR4 agonist lipopolysaccharide (LPS) induces TLR4 expression rather than that of TLR2 and results in elicitation of asthma. Thus, each TLR pathway could contribute distinctively to innate immunity in nose and lung mucosa [8].

Δ*pep27* is a non-toxic attenuated *pep27* mutant strain of pneumococcus. Previously, we reported the effect of the Δ*pep27* immunization in a mouse model of asthma in reducing Th2 related responses [9]. Moreover, Δ*pep27* relieved asthma symptoms by reducing serum IgE and Th2-related cytokine secretion in the lung [9]. Δ*pep27* was found to have non-invasive properties that inhibit autolysis and do not penetrate into the lungs, blood, or brain during infections in mice [10]. It has also been shown to provide serotype-independent protection that the currently available vaccines do not [11]. Thus, Δ*pep27* immunization has an anti-allergic and anti-inflammatory effect. Recently, the need for mucosal vaccines has become recognized. In particular, nasal mucosal vaccines have several advantages, including lack of needle injury, the convenience of vaccination, economic production, and induction of local immune responses [12-14]. However, it remains unknown whether the nasal Δ*pep27* immunization inhibits allergic response in the nasal cavity. Therefore, this study aims to determine whether Δ*pep27* can relieve allergic symptoms in the OVA-induced AR model by regulating the AR-related Th2 response, and whether it has the potential to be used as an AR-preventive vaccine.

## Materials and Methods

### Bacterial Strains

The THpep27 bacterial strain (Δ*pep27*) used in this work [15] was cultured at 37°C overnight on 5% sheep blood agar plates with 3% Todd-Hewitt broth (Difico Laboratories, BD, France) and 0.5% yeast extract (Difco Laboratories) and then grown in THY (3% Todd-Hewitt broth with 0.5% yeast) at 37°C. All media were sterilized by autoclaving at 121°C for 15 min.

### Animals

Five-week-old Female BALB/c mice (Orient, Korea) were maintained under specific pathogen-free conditions with a 12 h dark/light cycle at room temperature, and allowed food *ad libitum*. The Sungkyunkwan University Animal Ethical Committee approved the use of animals in this study following the Korean Animal Protection Law (SKKUIACUC2020-04-13-2).

### Δ*pep27* Immunization

Before developing nasal inflammation, mice received 1 × 10^8^ CFU of Δ*pep27* or PBS per animal to assess the preventive effect. Δ*pep27* was suspended in 50 μl of PBS and administered intranasally once a week for three weeks.

### OVA-Induced AR Model

**Fig. 1:**
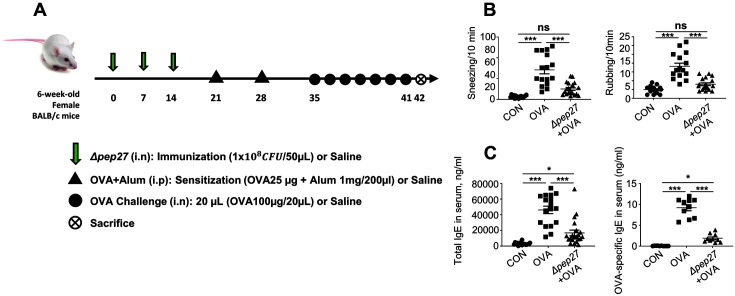
Δ*pep27* relieves AR symptoms, and represses total and OVA-specific IgE in an OVA-induced AR model. (**A**) Schematic diagram of the OVA-induced AR experiment using Δ*pep27* immunization. (**B**) The frequency of nasal rubbing and sneezing after the final challenge was assessed by counting for 10 min. (**C**) The total IgE and OVA-specific IgE levels in serum were determined by ELISA. Three independent experiments were performed, and the data are presented as the mean ± SEM, *p < 0.05, ***p < 0.001, ns; not significant. A representative of 3 independent experiments was analyzed with oneway ANOVA (Bonferroni’s Multiple Comparison Test).

Sensitizations were performed on days 0 and 7. Mice were sensitized intraperitoneally to ovalbumin (OVA: chicken egg albumin, grade V, Sigma-Aldrich, USA) absorbed with 2 mg aluminum hydroxide (Alum: Sigma-Aldrich, USA) in 100 μl saline (0.9% NaCl, Dynebio, Korea) for rhinitis, while the negative control group was treated with saline only. One week after the last sensitization, mice were challenged every day from day 35 to 41 by intranasal (I.N.) administration with 100 μg OVA in 20 μl saline or saline only (Fig. 1). The mice were euthanized with CO_2_.

### Nasal Symptom Scores and Sample Preparation

AR symptoms (sneezes and nasal rubbing) were observed for 10 min on day 41, immediately after the last OVA challenge. Mice were sacrificed after 24 h and serum was collected to measure IgE levels. The mouse nasal mucosa was carefully scraped off with a curette.

### Splenocyte Isolation

Mice were immunized with 1 × 10^8^ CFU of Δ*pep27* or PBS intranasally once a week for three weeks. After the last immunization, mice were sensitized with OVA once a week for two weeks, and one week after the last sensitization, mice were challenged with OVA once a day for three days. Spleens were harvested one day after the last OVA challenge and isolated splenocytes were treated with OVA (10 μg/ml) to stimulate Th2 responses. After 72 h incubation, culture media were harvested to determine cytokine levels.

### Determination of Total IgE and OVA-Specific IgE

Serum samples were collected 24 h after the last OVA challenge. Total IgE and OVA-specific IgE were determined using an enzyme-linked immunosorbent assay (ELISA) kit (Total IgE Mouse Uncoated ELISA kit, Invitrogen, USA, OVA-specific IgE, Legend Max, USA) according to the manufacturer’s instructions.

### Determination of Cytokine Levels

The splenocyte supernatant was analyzed for the concentration of IL-4 (#M4000B), IL-5 (#M5000), and IL-13 (#M1300CB) using ELISA kit (R&D system, USA) following the manufacturer’s instructions.

### Hematoxylin-Eosin (H&E) Staining

Nasal cavities were collected 24 h after the last OVA administration, fixed in 4% formalin solution, and then placed in a paraffin block. The tissue was embedded with paraffin and cut into 2 μm sections (KNOTUS, Korea), stained with hematoxylin-eosin, and then observed under an optical microscope (Olympus. BX53, Japan). The image was observed at 40X magnification. We prepared one slide per each mouse sample and examined them carefully. After evaluation of each slide, a representative slide per group was selected and used for statistical analysis.

### Real-Time qPCR

Total mRNA was extracted from the nasal mucosa using Trizol (Ambion, USA) and an EcoDry Premix kit (Takara, Japan) was used to synthesize complementary DNA (cDNA). qPCR was performed according to the manufacturer’s instructions (Applied Biosystems, USA) using the primers (Table 1). The amplification conditions were as follows: 95°C/15 sec, 40 cycles of 95°C/15 sec, 55°C/30 sec, and extension 72°C/30 sec; followed by melting curve analysis comprising 95°C for 15 sec, 60°C for 1 min, and 95°C for 15 sec.

### Protein Extraction and Western Blot

One day after the last OVA challenge, the nasal mucosa was gently scraped off with a curette and homogenized in a homogenizer (PRO Scientific Inc., Model 200 Double insulated, USA) in M-PER™ Mammalian Protein Extraction Reagent (Thermofisher, USA). Total protein concentrations were measured with a bicinchoninic acid assay (BCA) kit (Thermofisher). Protein samples were loaded onto SDS-PAGE using a 4-15% gradient gel and transferred to polyvinylidene fluoride membranes using Trans-Blot Turbo (Bio-Rad Laboratory, USA). After transfer, the membrane was blocked at room temperature with 5% skim milk in Tris-buffered saline with Tween-20 (TBS-T) and then probed with an appropriate antibody in TBS-T containing 5% skim milk overnight. Antibodies against TLR2 (#13744), TLR4 (#14358), p-IkBa (#2859), p-p65 (#3033), p65 (#8242), NLRP3 (#15101), caspase-1 (#24232), cleaved caspase-1 (#89332), and IL-1β (#12426) were from Cell Signaling Technology (USA), TLR5 (#ab62460) from Abcam (UK), TLR9 (#NBP2-24729) from Novus Biologicals (USA) and β-actin (#sc-47778) was from Santa Cruz Biotechnology (USA). The secondary antibody was an anti-mouse/rabbit immunoglobulin G antibody conjugated with horseradish peroxidase (HRP) with 5% skim milk in TBS-T, followed by detection using Clarity Max Western ECL Substrate (Bio-RAD with a Chemiluminescence Imaging System (FluorChem E., USA). To measure band intensity, AlphaView SA program was used.

### Statistical Analysis

Comparisons of symptoms score, eosinophil counts, cytokine levels, and IgE levels were analyzed with one-way analysis of variance (ANOVA) using Graph Pad Prism software (version 5, Graph Pad Software Inc, USA). Data are presented as an average of triplicate wells ± SEM. Statistically significant differences were defined as *, *p* < 0.05; **, *p* < 0.01; ***, *p* < 0.001 and ****, < 0.0001.

## Results

### Δ*pep27* Immunization Protects OVA-Induced AR

AR is commonly characterized by IgE-mediated hypersensitivity reactions such as sneezing and nasal itching [16]. OVA is used as an allergen test since it’s immunological effect on allergy is well characterized. To assess the effect of Δ*pep27* immunization on OVA-induced AR, a mouse model was established by intraperitoneal injection (sensitization) using OVA/alum and subsequent intranasal challenge with OVA (Fig. 1A). Allergic symptoms were scored by counting sneezing and rubbing in each group for 10 min after the last OVA challenge. In the normal control group, sneezing occurred 5 times and rubbing occurred 4 times, whereas, in the AR model, sneezing and rubbing were increased to 58 times and 15 times, respectively. In contrast, the number of sneezes in the immunized group was reduced significantly from 58 to 18, and the number of rubbings was also significantly reduced from 15 to 6 (Fig. 1B). The total IgE and OVA-specific IgE levels in serum were significantly increased in the OVA group. However, Δ*pep27* immunization significantly reduced both total IgE (78% less than the OVA group) and OVA-specific IgE (85% less than the OVA group) (Fig. 1C). Thus, Δ*pep27* immunization significantly alleviates AR symptoms and allergic IgE levels.

### Reduced Eosinophil and Th2 Responses by Δ*pep27*

**Fig. 2:**
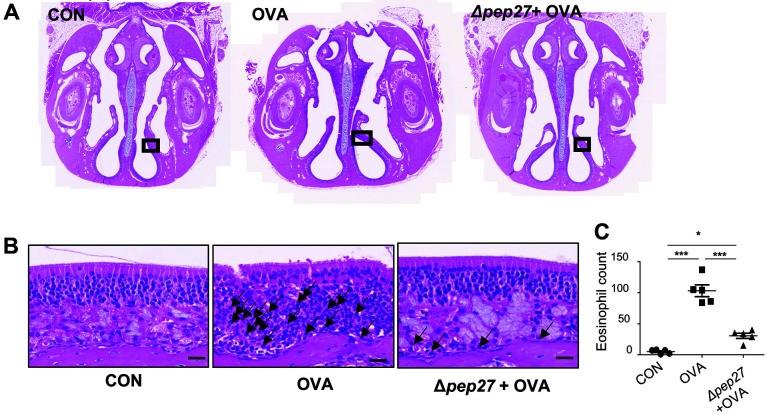
Δ*pep27* immunization suppresses eosinophil infiltration into the nasal mucosa. (**A**) H&E staining for eosinophils in the nasal cavity with 4X magnification. (**B**) H&E staining for eosinophils in (**A**) with the boxed region was shown with 40X magnification, scale bar = 20 μm. (**C**) The number of eosinophils in the nasal mucosa. P-value was calculated by one-way ANOVA and expressed as mean ± SEM, *p < 0.05, ***p < 0.001, ns; not significant (Bonferroni’s Multiple Comparison Test).

Eosinophil infiltration and the Th2 immune response have been proposed as one of several mechanisms underlying AR development and regulation [17]. Hematoxylin-eosin (H&E) staining was performed to investigate the inflammatory response in the nasal mucosa (Fig. 2A). This showed that in the AR model after OVA challenge, an average of 103 eosinophils was detected, compared to an average of 5 in the normal controls. Δ*pep27* immunization significantly reduced eosinophil penetration into the nasal turbinate mucosa by an average of 31 (Fig. 2B).

**Fig. 3:**
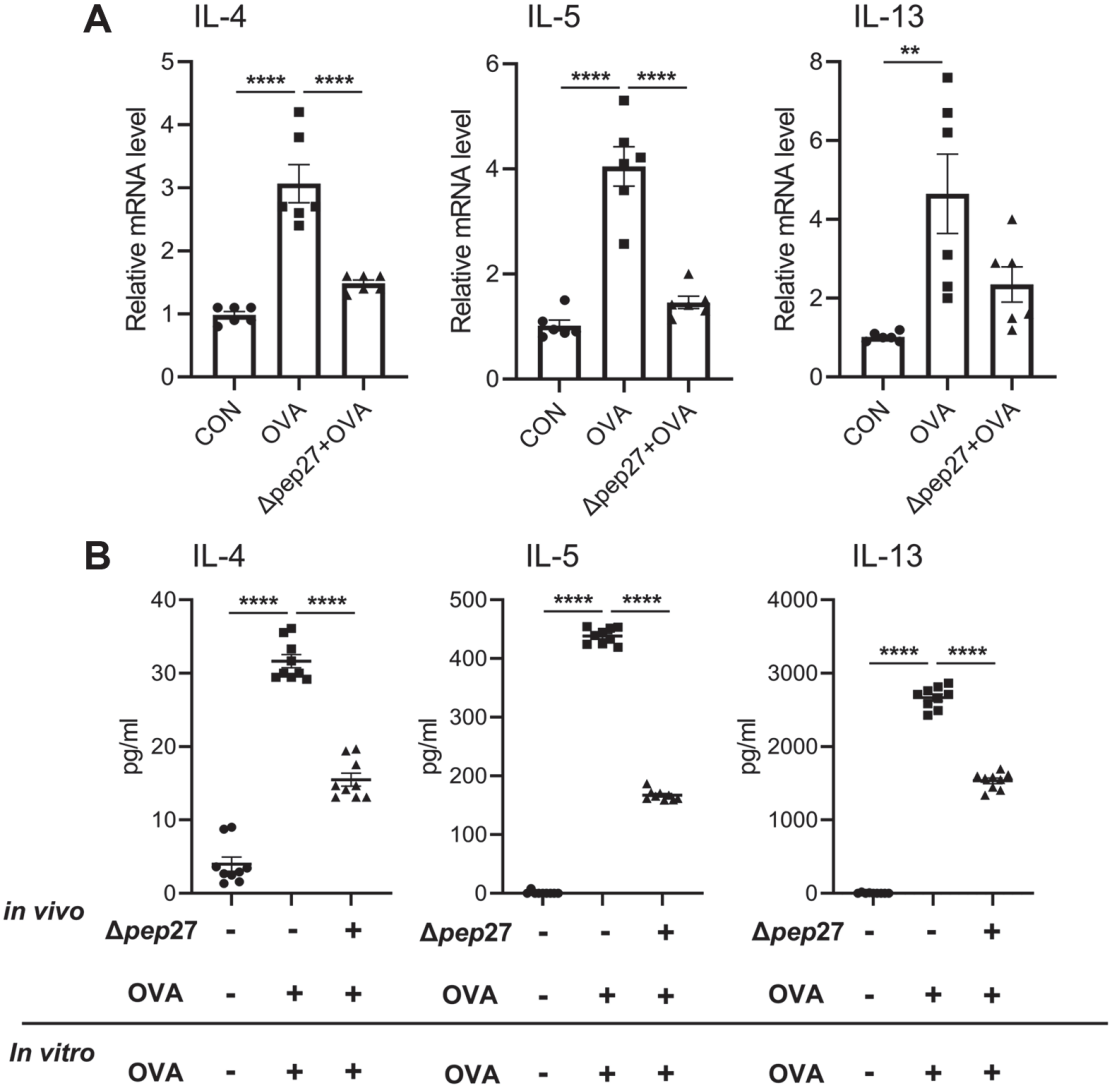
Δ*pep27* immunization inhibits Th2-dependent cytokines in the nasal mucosa. (**A**) Th2 cytokines in the nasal mucosa were determined by qPCR. (**B**) Production of Th2 cytokines in splenocyte supernatant was analyzed by ELISA. Values are presented as the mean ± SEMs (*n* = 9 per group). *p*-value was calculated by one-way ANOVA and expressed as mean ± SEM, **p < 0.01, ****p < 0.0001, ns; not significant (Tukey’s Multiple Comparison Test).

When transcripts of cytokines associated with AR in the nasal mucosa were measured by qPCR, Th2-dependent IL-4, IL-5, and IL-13 transcripts were significantly increased by OVA challenge compared to the normal control, while Δ*pep27* immunization decreased these transcripts by 50%, 65%, and 52%, respectively, compared to the OVA group (Fig. 3A).

To further corroborate Δ*pep27*-dependent Th2 inhibition in the spleen, IL-4, 5, and 13 levels in murine splenocytes were examined with or without Δ*pep27* immunization. When splenocytes were treated with OVA, Th2 cytokines significantly increased in the supernatant of splenocyte culture. However, when Δ*pep27* immunized splenocytes were challenged with OVA, Th2-related inflammatory cytokines were significantly reduced compared to those treated with OVA only (Fig. 3B), demonstrating that Δ*pep27* significantly reduces OVA-induced AR.

### Δ*pep27* Immunization Downregulates the Toll-Like Receptors Pathway.

**Fig. 4:**
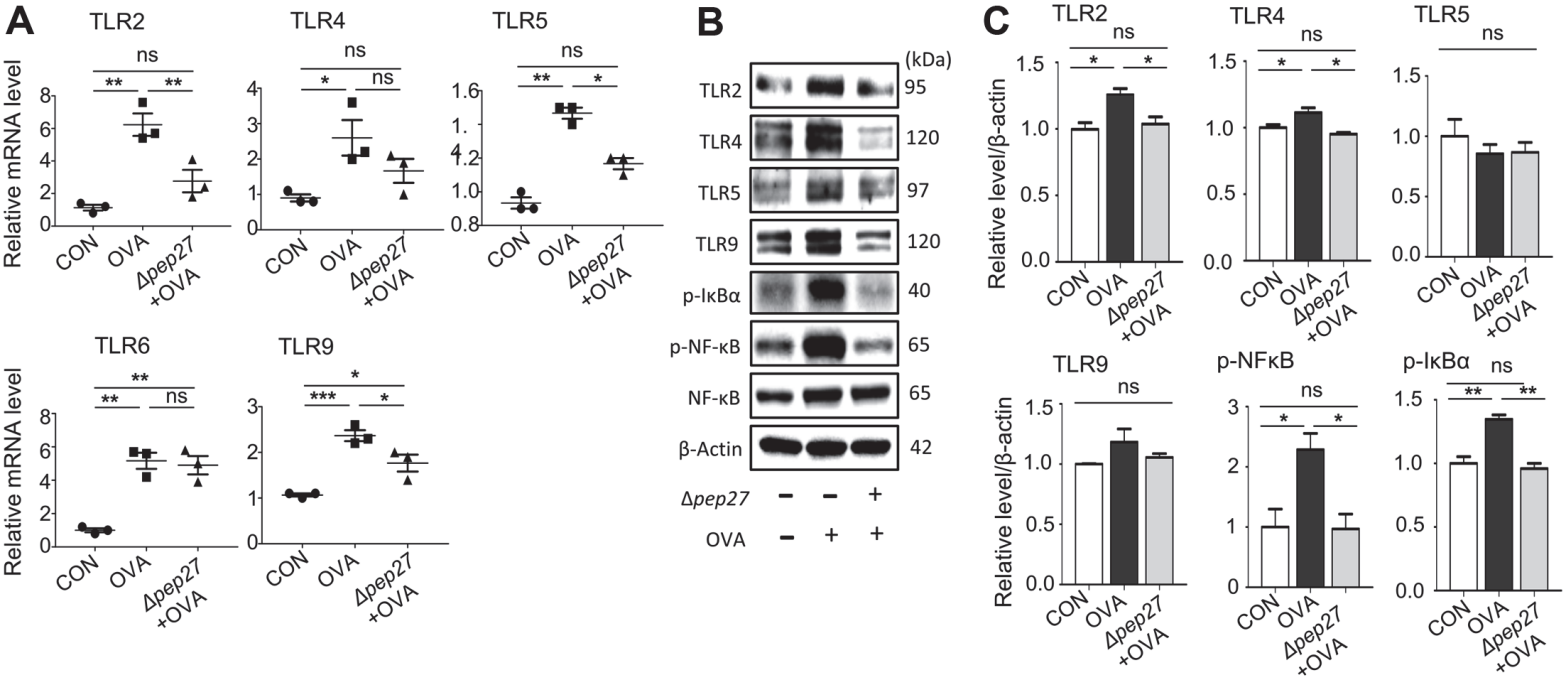
Δ*pep27* immunization represses the TLR pathway in the nasal mucosa. (**A**) mRNA and (B, C) protein levels were detected by qPCR and Western blot analysis, respectively. Values are presented as the mean ± SEMs (*n* = 3 per group), *p < 0.05, **p < 0.01, ***p < 0.001, ns; not significant (Tukey’s Multiple Comparison Test).

TLRs signaling is activated during AR development, and subsequently results in nuclear factor-κB (NF-κB) activation and inflammatory gene transcription [18]. In AR patients, TLR9 expression and IL-6 production were increased in basophils [19]. Moreover, TLR agonists with anti-allergic effects such as TLR4 and TLR9 agonists are under clinical trials. Other TLR agonists such as TLR2, TLR5, and TLR7 agonists have shown anti-allergic effects in animal studies [20]. Thus, to explore the underlying mechanism of Δ*pep27* immunization in AR prevention, nasal mucosa samples were collected for mRNA analysis. When TLR transcripts levels were quantified by qPCR, OVA treatment increased TLR transcription significantly, whereas Δ*pep27* decreased transcription of TLR 2, 5, and 9 significantly by 55%, 23%, and 17%, respectively, compared to the OVA control (Fig. 4A).

Western blot was used to further investigate TLR expression at the protein levels. OVA treatment significantly induced TLR expression, whereas Δ*pep27* immunization decreased TLR2 and 4 compared to the OVA control. However, the TLR5 level did not differ between the Δ*pep27* group and the OVA group. Furthermore, the protein level of NF-κB, which is activated by the TLR pathway, was significantly increased by OVA treatment, but the NF-κB signaling pathway was significantly repressed by the immunization (Fig. 4B). These results indicate that the TLR signaling pathway is downregulated by the Δ*pep27* immunization.

### Δ*pep27* Decreased NLRP3 Inflammasome Activation.

**Fig. 5:**
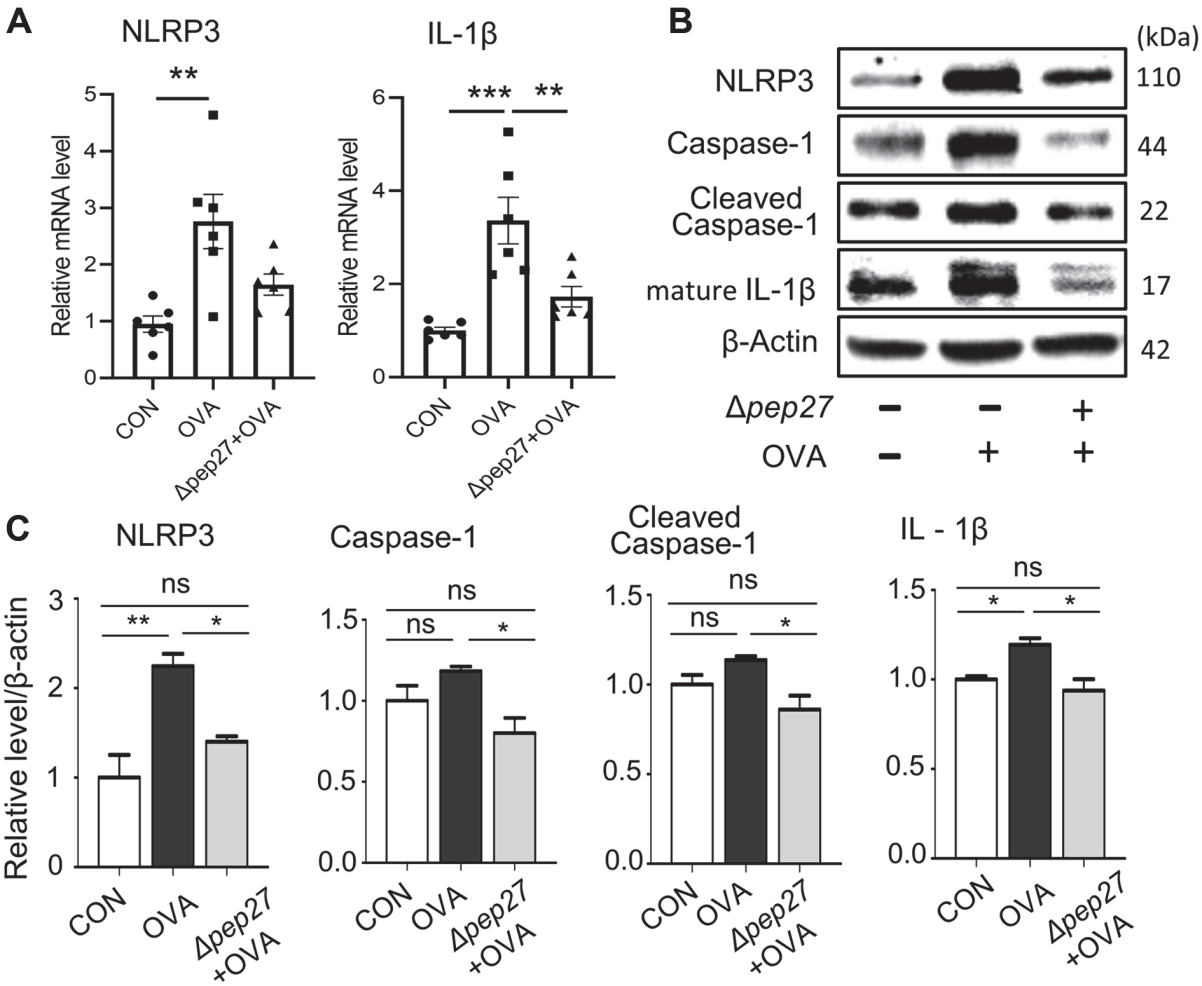
Δ*pep27* immunization inhibits NLRP3 inflammasome activation. (**A**) mRNA levels of NLRP3 and IL-1β in nasal mucosa were measured by qPCR. (**B**) Protein levels of NLRP3, caspase-1, cleaved caspase-1, p-IκB-α, and IL-1β in nasal mucosa were measured by Western blot (*n* = 6 per group). P-value was calculated by one-way ANOVA and expressed as mean ± SEM *p < 0.05, **p < 0.01 and ***p < 0.001, N.S; not significant (Tukey’s Multiple Comparison Test).

The NLRP3 inflammasome, which is composed of NLRP3, ASC (adaptor protein called apoptosis-associated speck-like protein containing a CARD), and pro-caspase-1, is currently the most extensively studied intracellular receptor, and its expression is increased upon TLR stimulation. In addition, NLRP3 inflammasome activation is increased in the nasal mucosa of both AR patients and AR mice [21]. Therefore, we investigated whether TLR repression by Δ*pep27* immunization can inhibit the NLRP3 inflammasome to reduce AR. When the mRNA level of NLRP3 in the nasal mucosa was measured by qPCR, the NLRP3 transcript was increased in OVA-induced mice compared to the normal control. However, the immunization decreased this to 40% of the OVA group (Fig. 5A). Since NLRP3 inflammasome activation increases inflammatory cytokines such as IL-1β [22], the mRNA level of IL-1β in the nasal mucosa was measured by qPCR. Results showed that IL-1β transcript was decreased by Δ*pep27* to 47% of the OVA group (Fig. 5A). In addition, when the protein levels of inflammasome-related factors were quantified by Western blot, NLRP3, caspase-1, cleaved caspase-1, and IL-1β levels were induced by OVA treatment, whereas these parameters were significantly decreased by Δ*pep27* immunization by 37%, 48%, 21.5%, 24%, respectively, in the OVA group (Fig. 5B) suggesting inhibition of NLRP3 inflammasome activation by Δ*pep27* immunization.

## Discussion

Mice immunized with Δ*pep27* before OVA exposure showed a significant decrease in sneezing and rubbing, serum total and OVA-specific IgE levels compared to mice exposed only to OVA. Histological analysis showed that Δ*pep27* intranasal immunization reduced eosinophil infiltration into the nasal mucosa. Thus, intranasal immunization of Δ*pep27* could successfully inhibit the development of AR.

Cell-mediated immunity is classified into 3 types: Type 1 immunity comprises T-bet^+^ IFN-γ-producing Th1 cell and mediates inflammation and autoimmunity. Type 2 immunity consists of GATA3^+^ Th2 cells and mediates allergy by producing IgE antibody as well as IL-4, IL-5, and IL-13. Type 3 immunity is composed of RORγt (retinoic acid-related orphan receptor γt+) Th17 cells and produces IL-17 and/or IL-22, which are involved in inflammation and autoimmunity [23]. IL-4 and IL-13 promote IgE production, and IL-5 induces eosinophil differentiation, activation, and survival [24, 25]. Therefore, down-regulation of these Th2 cytokines may reduce IgE secretion and ultimately ameliorate AR. In this study, Δ*pep27* immunization prior to allergen exposure significantly decreases Th2 cytokine secretion in the nasal mucosa when compared with the AR control. Additionally, in splenocyte supernatants, OVA challenge significantly increased protein levels of IL-4, 5, and 13 compared to the control group, whereas Δ*pep27* immunization mitigated this induction.

Activation of some TLRs results in sensitizations and disruption of tolerance, but activation of some members of this family may promote tolerance to harmless allergens [26]. TLR is a new and promising target for allergen immunotherapy. Several studies have shown an association between TLRs and AR. When the TLR signaling pathway is activated in OVA-induced allergic inflammation mice, pro-inflammatory cytokines such as IL-1β are secreted by the induced NF-κB [27, 28]. Some TLR agonists decrease Th2 responses and relieve allergic diseases; for instance, TLR2 and TLR4 agonists can alleviate asthma symptoms [26, 29]. On the other hand, some TLR4 agonists, such as lipopolysaccharide (LPS), make the disease worse [30]. Moreover, TLR2, 6, and 9 agonists given before allergen challenge markedly inhibited early and late phase reactions of allergic diseases [31-33]. Intranasal administration of TLR7 [26] and TLR8 [34] agonists improved AR as therapeutics. Several receptors on immune cells respond to bacterial invasion by recognizing bacterial cell wall components. TLR2 recognizes peptidoglycan, lipopeptides, and lipoteichoic acid from *S. pneumoniae* [35, 36]. TLR4 recognizes phosphorylcholine and the exotoxin pneumolysin in *S. pneumoniae* [37, 38], although the results are conflicting. In addition, TLR2 and TLR4 are known to be involved in controlling *S. pneumoniae* infection and play a partially overlapping role. A recent study by us and others showed that a component of *S. pneumoniae* worked as an immunoregulatory therapy for allergic diseases. Intranasal immunization with Δ*pep27* reduced inflammatory cytokine secretion and serum IgE in the lung of the OVA-induced asthma model [9]. Moreover, pneumococcal protein conjugate vaccine (PCV) and pneumococcal polysaccharide vaccine (PPV) administered before or after allergen sensitization effectively inhibited the allergen-specific Th2 response and enhanced induction of Treg cells in an in vivo model of AR [39]. Our results revealed that OVA challenge significantly increased TLR2, 4, 5, and 9 transcription in the nasal mucosa, whereas Δ*pep27* immunization decreased TLR2, 5, and 9 transcripts. TLR2 and 4 protein levels in the nasal mucosa of the immunized group were significantly reduced compared to those of the OVA group. Furthermore, the protein level of NF-κB elicited during the TLR activation was also significantly reduced. These results indicate that TLR inhibition by Δ*pep27* mainly alleviates NLRP3 inflammasome activation and contributes to mitigate OVA-induced AR. These results suggest that immune tolerance can be elicited by attenuated pneumococcal components, and some components of Δ*pep27* might inhibit TLR activation by inducing immune tolerance on the nasal mucosa.

NF-κB also has a role in regulating the activation of inflammasomes [40], as NF-κB signaling activation upregulates the expression of the inflammasome component NLRP3 and pro-inflammatory cytokines [41]. Once activated, NLRP3 oligomerizes and recruits an ASC, forming a complex and activation of the caspase-1 protease. When NLRP3 inflammasome was activated, caspase-1 cleaves the pro-inflammatory cytokines such as IL-1β and IL-18 [42], mediating the secretion of inflammatory cytokines [43]. Thus, NLRP3 seems to play some role in the development of AR, and may be a target for AR therapy. An interaction between TLRs and NLRP3 was observed upon Δ*pep27* immunization. Our results indicate that the Δ*pep27* immunization markedly reduced NLRP3 and IL-1β expression at mRNA and protein levels. In addition, in the nasal mucosa, both pro-caspase-1 and cleaved caspase-1 were reduced by Δ*pep27* immunization. Based on these studies, NLRP3 protein seems to play an important role in Th2 mediated OVA-induced AR. Exposure of type 2 cells to IL-1β enhances Th2 differentiation at the early stage and induces IL-13 gene transcription in Th2 cells but decreases IL-4 differentially, resulting in induction of inflammatory Th2 response [44, 45]. Thus, inhibition of TLR signaling followed by lower IL-1β level can result in attenuated IL-1β priming and subsequent reduction of allergic response.

Moreover, we demonstrated already that Δ*pep27* immunization could elicit Th1, Th17, and Treg upregulation but Th2 downregulation, whereas OVA-induced asthma model showed downregulation of Th1, Th17, and Treg and upregulation of Th2 response [9].

TLR activation leads to pro-inflammatory responses [46]. In OVA-induced asthma model, Δ*pep27* immunization induces anti-inflammatory Treg transcription factor Foxp3, and represses allergic Th2 transcription factor (GATA-3). Subsequently, Δ*pep27* immunization represses Th2 specific allergic cytokines such as IL-4, IL-5, and IL-13 in the bronchoalveolar lavage fluid (BALF). Moreover, Δ*pep27* immunization inhibits secretion of inflammatory cytokine TNF-α thus helps to maintain homeotic milieu during allergic environment [9]. Thus, Δ*pep27* immunization seems to induce anti-inflammatory Treg and subsequently repress inflammatory TLR activation and result in attenuation of allergic IgE as well as Th2 specific cytokines.

**Fig. 6:**
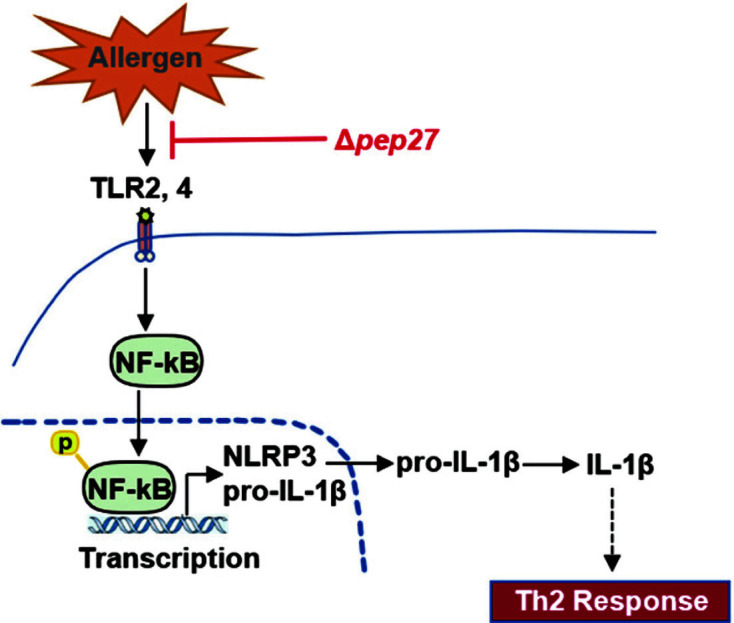
Δ*pep27* immunization negatively regulates NLRP3 inflammasome activation. NF-κB is translocated to the nucleus of immune cells through activation of TLR during AR and then activates transcription of NLRP3. Immunization with Δ*pep27* in the nasal mucosa reduces the Th2 response.

Collectively, pneumococcal Δ*pep27* can downregulate TLR2 and TLR4, Th2 cytokines, and inflammatory cell infiltration in the nasal mucosa, thus suppressing NF-κB activation and NLRP3 inflammasome activation in the nasal mucosa possibly via repressed Th2 responses (Fig. 6). Together, our results demonstrate that Δ*pep27* intranasal immunization could be used as a mucosal vaccine in patients with AR.

## Figures and Tables

**Table 1:** The gene-specific primers used in this study.

Gene	Primer sequence (5’→3’)
IL-4	5’ -AGATGGATGTGCCAAACGTCCTCA -3’ 5’ -AATATGCGAAGCACCTTGGAAGCC-3’
IL-5	5’ -GCTTCTGCACTTGAGTGTTCTG-3’ 5’ -CCTCATCGTCTCATTGCTTGTC-3’
IL-13	5’ -TGAGGAGCTGAGCAACATCACACA-3’ 5’ -TGCGGTTACAGAGGCCATGCAATA-3’
IL-1β	5’-TGTGAAATGCCACCTTTTGA-3’ 5’-GTGCTCATGTCCTCATCCTG-3’
TLR2	5’-ACAGCAAGGTCTTCCTGGTTCC-3’ 5’-GCTCCCTTACAGGCTGAGTTCT-3’
TLR4	5’-TGGCTGGTTTACACATCCATCGGT-3’ 5’-TGGCACCATTGAAGCTGAGGTCTA-3’
TLR5	5’-AGCATTCTCATCGTGGTGG-3’ 5’-AATGGTTGCTATGGTTCGC-3’
TLR6	5’ -TGGATGTCTCACACAATCGG-3’ 5’ -GCAGCTTAGATGCAAGTGAGC-3’
TLR9	5’ -TATCCACCACCTGCACAACT-3’ 5’ -TTCAGCTCCTCCAGTGTACG-3’
NLRP3	5’ -TGCTCTTCACTGCTATCAAGCCCT-3’ 5’ -ACAAGCCTTTGCTCCAGACCCTAT-3’
GAPDH	5’-TCAACAGCAACTCCCACTCTTCCA-3’ 5’-ACCCTGTTGCTGTAGCCGTATTCA-3’
